# Comparative Metabolite Profiling of *Betula pendula* Roth and *Betula pubescens* Ehrh. Leaf Extracts Reveals Antioxidant and Hepatoprotective Potential

**DOI:** 10.3390/molecules31173037

**Published:** 2026-08-28

**Authors:** Timea Henrietta Dezső (Bab), Irina Ielciu, Alina Diana Hașaș, Bogdan Sevastre, Neli Kinga Olah, Emoke Pall, Raluca Marica, Alexandra-Cristina Sevastre-Berghian, Daniela Benedec, Ilioara Oniga, Ramona Păltinean, Daniela Hanganu

**Affiliations:** 1 Department of Pharmacognosy, Faculty of Pharmacy, “Iuliu Hațieganu” University of Medicine and Pharmacy, 400010 Cluj-Napoca, Romaniadbenedec@umfcluj.ro (D.B.); ioniga@umfcluj.ro (I.O.); dhanganu@umfcluj.ro (D.H.); 2 Department of Pharmaceutical Botany, Faculty of Pharmacy, “Iuliu Hațieganu” University of Medicine and Pharmacy, 400337 Cluj-Napoca, Romania; rpaltinean@umfcluj.ro; 3 Faculty of Veterinary Medicine, University of Agricultural Sciences and Veterinary Medicine, 400372 Cluj-Napoca, Romania; alina.hasas@usamvcluj.ro (A.D.H.); bogdan.sevastre@usamvcluj.ro (B.S.); raluca.marica@usamvcluj.ro (R.M.); 4 Department of Medical Chemistry, Faculty of Pharmacy, “Vasile Goldiș” Western University of Arad, 310414 Arad, Romania; olah.neli@uvvg.ro; 5 PlantExtrakt Ltd., 407059 Cluj-Napoca, Romania; 6 Department of Clinical Sciences, Faculty of Veterinary Medicine, University of Agricultural Sciences and Veterinary Medicine, 400372 Cluj-Napoca, Romania; emoke.pall@usamvcluj.ro; 7 Department of Physiology, Faculty of Medicine, “Iuliu Hațieganu” University of Medicine and Pharmacy, 400012 Cluj-Napoca, Romania; alexandra_berghian@yahoo.com

**Keywords:** silver birch, downy birch, phytochemical analysis, cytotoxic, in vitro and in vivo biological effects, hepatoprotective

## Abstract

The present study compares the polyphenolic composition, as well as the antioxidant and hepatoprotective potential of extracts obtained from the leaves of *Betula pendula* Roth and *Betula pubescens* Ehrh. The extracts were prepared using 90% *v*/*v* ethanol and the phytochemical contents were assessed using spectrophotometric assays, while independent metabolites were identified and quantified using an LC/MS/ESI method. The antioxidant potential was evaluated by the DPPH radical and FRAP scavenging assays. Cytotoxicity was evaluated using human LX-2 hepatic stellate cells and HepG2 hepatocellular carcinoma cells. Cell viability assays were performed to determine the potential cytotoxic effects of the two extracts on cellular viability and overall metabolic activity. In vivo, hepatoprotective potential was assessed in a CCl_4_-induced model of hepatotoxicity. Results revealed the presence of flavonoids and polyphenolic compounds in the leaves of both species, with a more significant potential for the *B. pendula* leaf extracts. The cell viability assays showed that both extracts maintained high cell viability in LX-2 cells, whereas *B. pubescens* induced a more pronounced reduction in HepG2 cell viability than *B. pendula* under the tested experimental conditions. In vivo, CCl_4_ increased ALT activity to 193.0 ± 8.5 U/L, whereas *B. pendula* and *B. pubescens* at 200 mg/kg reduced ALT to 30.4 ± 6.6 and 117.4 ± 24.3 U/L, respectively. CCl_4_ also increased hepatic MDA levels from 202.7 ± 31.8 to 717.1 ± 31.6, which were reduced to 430.7 ± 100.5 and 448.7 ± 65.8 following treatment with *B. pendula* and *B. pubescens* at 200 mg/kg, respectively.

## 1. Introduction

*Betula* L. (birches) represents the largest genus within the Betulaceae family, encompassing more than 100 recognized species distributed globally across temperate and boreal ecosystems of the northern hemisphere [1]. In many countries of northern and western Europe, birch constitutes the primary source of hardwood [2,3]. The genus consists predominantly of trees and shrubs that are monoecious, bearing simple, alternate, deciduous, toothed or serrate leaves; male flowers are arranged in pendulous spikes, while female flowers occur in either erect or pendulous spikes [1,4]. *Betula pendula* Roth (silver birch) and *Betula pubescens* Ehrh. (downy birch) are the most ecologically dominant and geographically widespread species across Europe, with a combined range extending from the Atlantic coast through to western Siberia and beyond. Owing to this extensive distribution, both species exhibit considerable morphological variability, and numerous subspecies and varieties have been formally described [5]. Furthermore, natural hybridization between the two species occurs throughout much of Europe, giving rise to forms with intermediate morphological characteristics. Birches are also capable of producing polyploid forms, and this tendency, combined with their inherent variability, interspecific hybridization, and the more recent introduction of artificially propagated cultivars beyond their native ranges, substantially complicates both their identification and the broader taxonomic classification of the genus *Betula* [2,6]. In mature specimens of young trees, the bark is silvery-white and bears horizontal dark gray lenticels that deepen in color and develop fissures over time. The bark of *B. pendula* is notably brighter and more lustrous than that of *B. pubescens*, and its branches are characteristically pendant, in contrast to those of *B. pubescens*, which grow in an upward or horizontal orientation. Additionally, the shoots of *B. pubescens* are covered by a fine, soft pubescence, whereas those of *B. pendula* are glabrous [1,2,4]. Their ecological role as pioneer species, their significant contribution to biodiversity, and their diverse traditional uses make birches subjects of considerable scientific interest [3].

From an ethnomedicinal perspective, birch has been used across a wide range of cultures throughout the Northern Hemisphere. The bark, leaves, sap, and buds have all been used to prepare traditional remedies in Russian, Scandinavian, German, and Eastern European folk medicine traditions, with applications spanning diuretic, cholagogue, anti-inflammatory, dermatological, and antimicrobial uses [1,7]. The leaf-derived botanical drug *Betulae folium* is among the most thoroughly characterized botanical phytomedicinal preparations today, holding monograph status in both the European Pharmacopeia and the British Pharmacopeia, and receiving endorsement from both the European Medicines Agency (EMA) and the European Scientific Cooperative on Phytotherapy (ESCOP) [8].

The distribution and chemical forms of flavonoids vary across different organs of *Betula* sp. In the leaves, flavonoids are predominantly present in glycosidic form, whereas the buds are characterized by a high concentration of aglycones, principally quercetin, naringenin, apigenin, and kaempferol [9,10]. More specifically, the leaves of both *B. pendula* and *B. pubescens* contain diverse phenolic profiles dominated by flavonoid glycosides, hydroxycinnamic acids, condensed tannins (proanthocyanidins and ellagitannins), and additional phenolic constituents such as flavonols and diarylheptanoids, with documented variation according to season and developmental stage [11,12]. Targeted evidence identifies specific flavonoid glycosides—including hyperoside and derivatives of quercetin and kaempferol—as well as hydroxycinnamic acids such as chlorogenic acid, collectively establishing a polyphenol-rich chemical baseline for the leaves of both species [13,14]. The leaf polyphenolic spectrum is further broadened, within the wider *Betula* spp. literature, by the presence of diarylheptanoids and lignans, with glycosylated flavonoids and catechins being particularly prominent among the low-molecular-weight phenolic compounds detected during both leaf development and at full maturity. Taken together, these findings support a polyphenol-dominated leaf profile across the two species [15]. Nevertheless, ongoing debate in the literature surrounds the question of which individual compounds are most chemotaxonomically diagnostic for *Betula* spp. leaves, and the extent to which specific polyphenol classes correlate with observed bioactivities. Although several studies highlight the predominance of flavonoid glycosides and tannins in *Betula* spp. leaves, the precise chemotaxonomic markers that distinguish *B. pendula* from *B. pubescens* remain inconsistently defined and are likely influenced by genotype, geographical location, and seasonal factors [13,15].

Research into the hepatoprotective properties of *Betula* species remains limited, with investigations focused almost exclusively on isolated compounds or fractions derived from the bark and leaves. Bark-derived triterpenoids—mainly betulinic acid and betulin, obtained primarily from *B. platyphylla*, *B. utilis*, and *B. pubescens*—have been evaluated in ethanol-, CCl_4_-, D-galactosamine-, and LPS-induced hepatotoxicity models in rodents, demonstrating notable hepatoprotective activity through modulation of oxidative stress markers (SOD, GSH, MDA), inflammatory pathways (NF-κB, TNF-α, TLR4), and fibrotic signaling axes (JAK/STAT, SOCS1) [16,17,18,19,20]. The sole available clinical evidence comes from a pilot study conducted by Shikov et al., in which a standardized birch bark extract containing 75% betulin resulted in ALT normalization and a reduction in HCV RNA levels in patients with chronic hepatitis C over a 12-week treatment period [21]. In contrast, the leaves, which are the officinal botanical drug, present a distinct and complementary phytochemical profile, dominated by flavonoid glycosides—including hyperoside, isoquercitrin, myricitrin, and quercetin glucuronide—which are themselves well-documented hepatoprotective agents. Nonetheless, no clear consensus exists regarding which specific leaf polyphenols of *Betula* spp. are the principal contributors to hepatoprotection, nor whether particular glycosides (such as myricetin glycosides or quercetin derivatives), hydroxycinnamic acids, or polymeric tannins bear the greatest responsibility, or how differences in their relative abundance between *B. pendula* and *B. pubescens* may influence the magnitude of hepatoprotective activity. Indeed, to the best of our knowledge, no study to date has undertaken a systematic comparison of leaf extracts from both *B. pendula* and *B. pubescens* within the same experimental model. Given the sympatric distribution of these two species across Europe—including Romania—a comparative investigation of their phytochemical profiles and bioactivities would constitute a genuinely novel and practically relevant contribution to the field.

In this context, the present study aims to evaluate the hepatoprotective activity of *Betula pendula* Roth (silver birch) and *Betula pubescens* Ehrh. (downy birch) leaf extracts and to connect it to the polyphenolic composition. The novelty of the present study relies on intersecting gaps. First, it is the first study that uses this approach to evaluate the hepatoprotective activity of *Betula* spp. leaf extracts. Moreover, no study has run a side-by-side phytochemical and hepatoprotective comparison of leaves from both species using the same methodology and the same experimental model. This comparative design generates chemotaxonomic data on whether the two species are interchangeable pharmacopoeially, which has direct future regulatory and therapeutic implications, opening new directions for the uses of leaves.

## 2. Results

### 2.1. Determination of TPC, FC, CADC and Antioxidant Activity in Betula spp. Extracts

The results of spectrophotometric analyses of polyphenolic compounds (TPC in mg GAE/g dw, TFC in mg RE/d dw and CADC in mg CAE/g dw), together with the antioxidant activity evaluated by DPPH and FRAP assays for the two *Betula* spp. extracts are presented in Table 1 Following quantification, it was found that the concentration of polyphenolic compounds differed between the two birch extracts: the *B. pendula* sample presented a higher content of total polyphenols (TPC 39.5 mg GAE/g dw), while the *B. pubescens* extract was richer in flavonoid and phenylpropane compounds (TFC: 29.7 mg RE/g dw and CADC: 11.1 mg CAE/g dw, respectively). The *B. pendula* extract presented a higher TPC value (39.5 ± 0.07 mg GAE/g dw) compared to the *B. pubescens* sample (31.1 ± 0.19 mg GAE/g dw), with statistically significant differences between samples (*p* < 0.05). The *B. pubescens* extract recorded the highest TFC value (29.7 ± 0.02 mg RE/g dw), significantly higher compared to the *B. pendula* leaf extract (10.6 ± 0.044 mg RE/g dw) (*p* < 0.05). Regarding CADC, the values determined were close between the two samples (11.1 ± 0.006 mg CAE/g dw for *B. pubescens* and 10.8 ± 0.08 mg CAE/g dw for *B. pendula*), and the differences were not statistically significant (*p* > 0.05). The *B. pendula* extract exhibited significantly higher antioxidant potential than *B. pubescens* extract, as demonstrated by a lower DPPH IC_50_ (0.23 ± 0.06 mg/mL vs. 0.69 ± 0.51 mg/mL, *p* < 0.05) and higher FRAP values (1.784 ± 0.073 mM TE/g dw vs. 1.078 ± 0.08 mM TE/g dw, *p* < 0.05) (Table 1). Both samples showed a higher IC_50_ than Trolox, indicating a significantly lower antioxidant activity compared to Trolox (*p* < 0.001).

### 2.2. Phytochemical Profile

The LC/MS/ESI analysis revealed a complex polyphenolic composition in the leaf extracts of *B. pendula* and *B. pubescens*, characterized by the presence of both phenolic acids and flavonoids (Table 2). The main phenolic metabolite was isoquercitrin, for both species. Interestingly, quercitrin was found in significant amounts only in the composition of *B. pubescens* leaves extract. Another flavonoid, hyperoside, was also found in important amounts in the leaves of both species. Among phenolic acids, cryptochlorogenic acid was the main metabolite. Overall, significant differences were noticed between composition of the two species, with *B. pubescens* proving to be the sample having more important amounts in most cases.

### 2.3. Cytotoxicity Results

The effects of leaf extracts obtained from *B. pubescens* and *B. pendula* on cell viability were evaluated in HepG2 cells at five different concentrations. The tested concentrations were selected based on the total polyphenol content of the extracts. Cell viability was expressed as a percentage relative to untreated control cells, considered as 100% viability.

**Figure 1:**
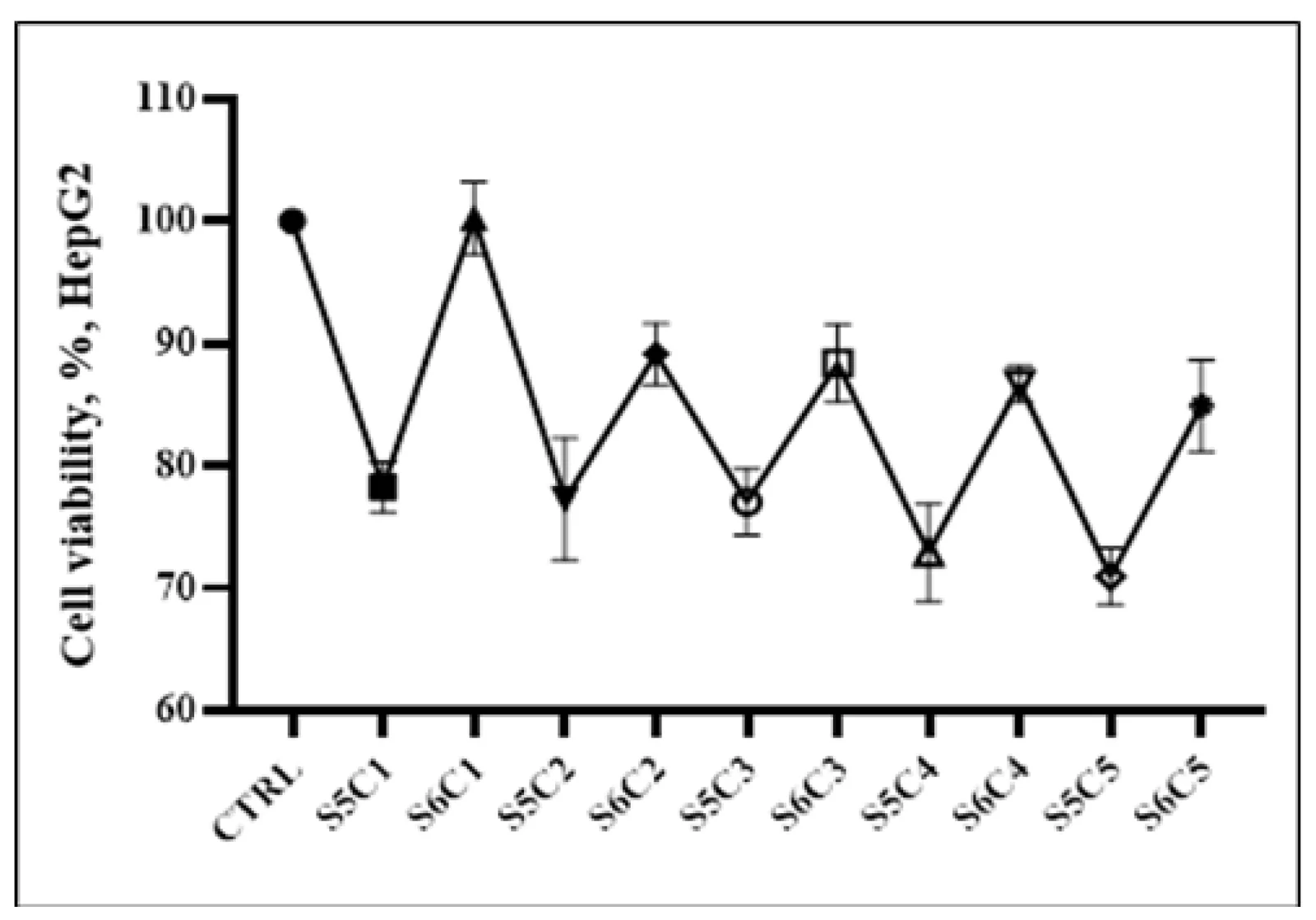
Effect of *Betula pendula* and *Betula pubescens* leaf extracts on the viability of HepG2 hepatocellular carcinoma cells. S5 and S6 represent *B. pubescens* and *B. pendula*, respectively, while C1–C5 indicate the five tested concentrations. Cell viability was determined using the CCK-8 assay after 24 h of treatment and expressed as a percentage relative to untreated control cells (100%). Data are presented as mean ± SD (n = 3).

In HepG2 cells, the *B. pubescens* extract induced a moderate reduction in cell viability across the tested concentration range, with mean viability values ranging from approximately 71% to 78%. In contrast, *B. pendula* extract exhibited a weaker effect, with mean viability values ranging from approximately 85% to 100% (Figure 1). Thus, *B. pubescens* induced a more pronounced reduction in HepG2 cell viability than *B. pendula* under the tested experimental conditions. The observed difference may be related to variations in the phytochemical composition of the extracts; however, the underlying molecular mechanisms were not investigated in the present study. Cell viability did not decrease to 50% at any of the tested concentrations. Consequently, an experimental IC_50_ value could not be determined within the investigated concentration range for either extract.

**Figure 2:**
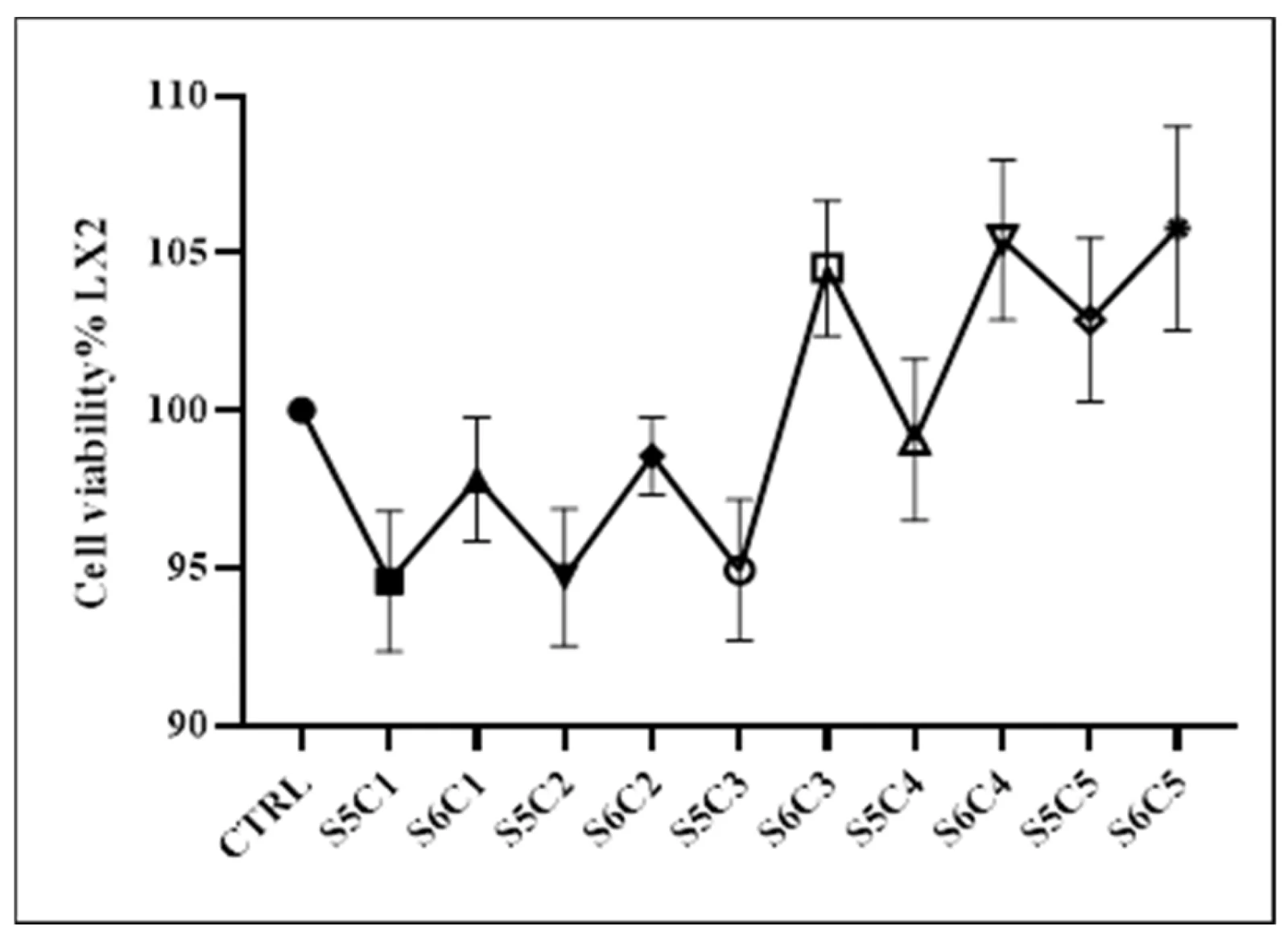
Effect of *Betula pendula* and *Betula pubescens* leaf extracts on the viability of LX-2 hepatic stellate cells. S5 and S6 represent *B. pubescens* and *B. pendula*, respectively, while C1–C5 indicate the five tested concentrations. Cell viability was determined using the CCK-8 assay after 24 h of treatment and expressed as a percentage relative to untreated control cells (100%). Data are presented as mean ± SD (n = 3).

In LX-2 hepatic stellate cells, both extracts maintained high cell viability across all tested concentrations. Treatment with *B. pubescens* resulted in mean viability values ranging from approximately 95% to 103%, whereas *B. pendula* extract maintained mean viability between approximately 98% and 106%. In several cases, viability values slightly exceeded the control level, which may reflect variations in cellular metabolic activity. However, the CCK-8 assay alone does not allow these changes to be attributed to increased cell proliferation or a specific stimulatory or protective effect. No concentration tested resulted in a 50% reduction in cell viability. Therefore, the IC_50_ values for both extracts were above the highest concentrations tested in LX-2 cells (Figure 2).

Overall, these findings indicate that *B. pubescens* induced a more pronounced reduction in HepG2 cell viability than *B. pendula* under the tested experimental conditions, whereas both extracts maintained high cell viability in LX-2 cells. Because the concentration ranges were normalized according to the total phenolic content of each extract, the C1–C5 positions do not represent identical nominal concentrations between *B. pendula* and *B. pubescens*. Therefore, comparisons between the two extracts at the same C1–C5 position should be interpreted with caution and should not be considered direct comparisons at equivalent absolute doses. The absence of a 50% reduction in cell viability within the tested concentration ranges indicates that the IC_50_ values were not reached under the experimental conditions used (Table 3).

### 2.4. Hepatoprotective Activity

Body weight was monitored throughout the 6-week experimental period. Minor fluctuations were observed between groups during the study; however, statistical analysis revealed no significant differences in body weight at any evaluated time point (*p* > 0.05). Overall, neither CCl_4_ administration nor treatment with chlorogenic acid or *Betula* spp. extracts produced clinically relevant changes in body weight, suggesting that the experimental protocol had no major impact on the body weight and body scoring condition of the animals.

**Figure 3:**
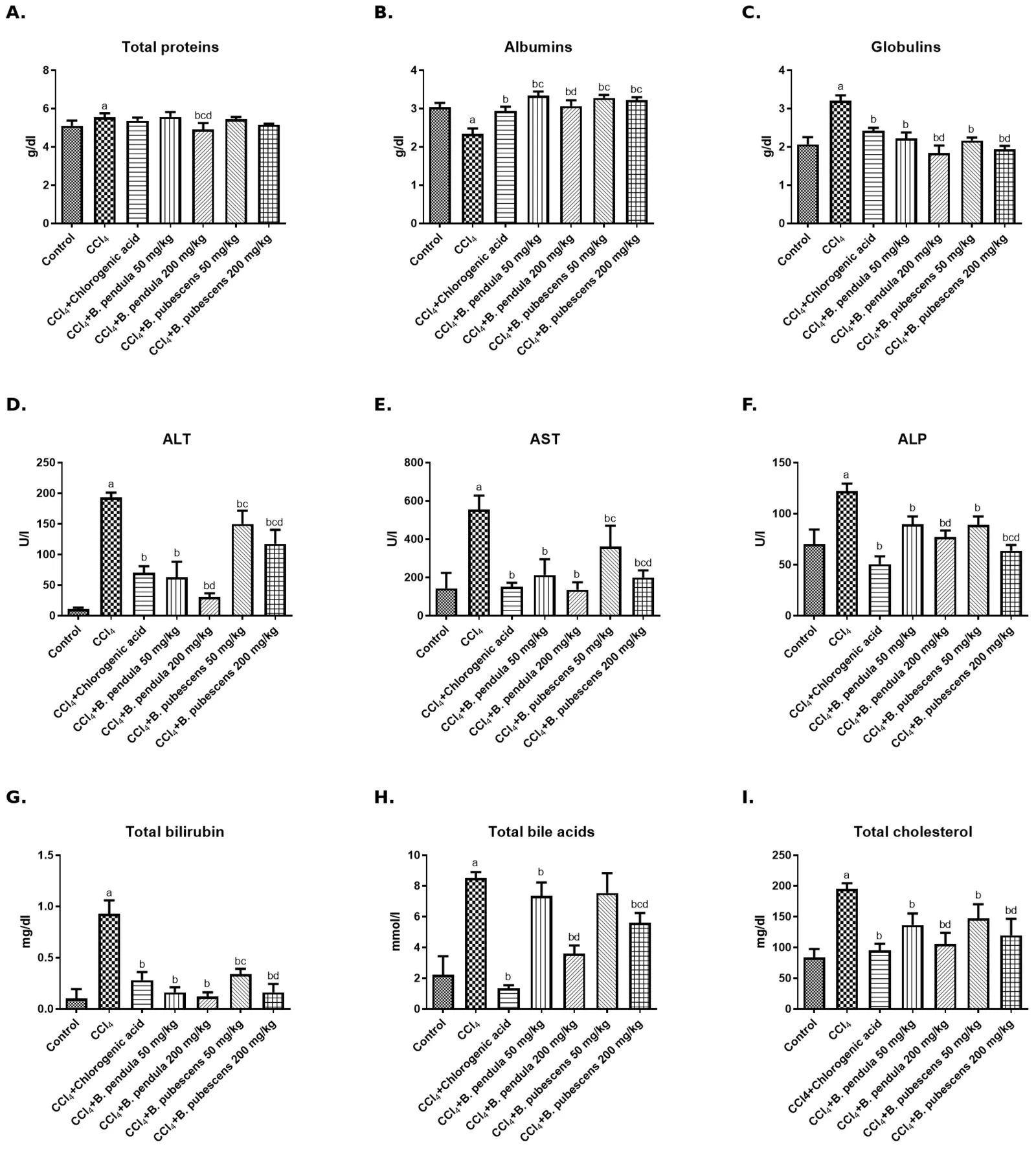
Effects of chlorogenic acid and *Betula* spp. extracts on: total proteins (**A**); albumins (**B**); globulins (**C**); ALT (**D**); AST (**E**); ALP (**F**); total bilirubin (**G**); total bile acids (**H**); and total cholesterol (**I**) (mean ± SD, 5 animals/group). a—*p* < 0.05 compared to the control group; b—*p* < 0.05 compared to the CCl_4_ group; c—*p* < 0.05 compared to the CCl_4_ + chlorogenic acid group; d—*p* < 0.05 between different doses of the same extract.

The effects of chlorogenic acid and *Betula* spp. extracts on serum biochemical parameters in CCl_4_-induced hepatotoxicity are presented in (Figure 3). Administration of CCl_4_ resulted in marked alterations of hepatocellular injury and cholestatic biochemical markers compared to the control group. Hepatocellular injury was characterized by increased alanine aminotransferase (ALT) and aspartate aminotransferase (AST) activities, both treatments, with chlorogenic acid and *Betula* spp. extracts, led to a reduction in these parameters compared to the toxic control group, with more pronounced effects observed at higher doses.

In addition, cholestasis was evidenced by elevated alkaline phosphatase (ALP) activity, total bilirubin, total bile acids, and cholesterol levels. Administration of chlorogenic acid and *Betula* spp. extracts reduced these parameters compared to the toxic control group, suggesting an improvement in hepatic excretory function. Overall, higher doses of *Betula* spp. extracts (200 mg/kg) exhibited a more pronounced effect compared to lower doses (50 mg/kg).

The administration of CCl_4_ significantly degraded the hepatic synthetic capacity, leading to a suppression of albumin production, while globulin synthesis was increased. Treatment with chlorogenic acid and *Betula* spp. extracts resulted in a partial normalization of these parameters, with albumin levels showing a tendency to increase total protein and globulin values approaching those of the control group.

**Figure 4:**
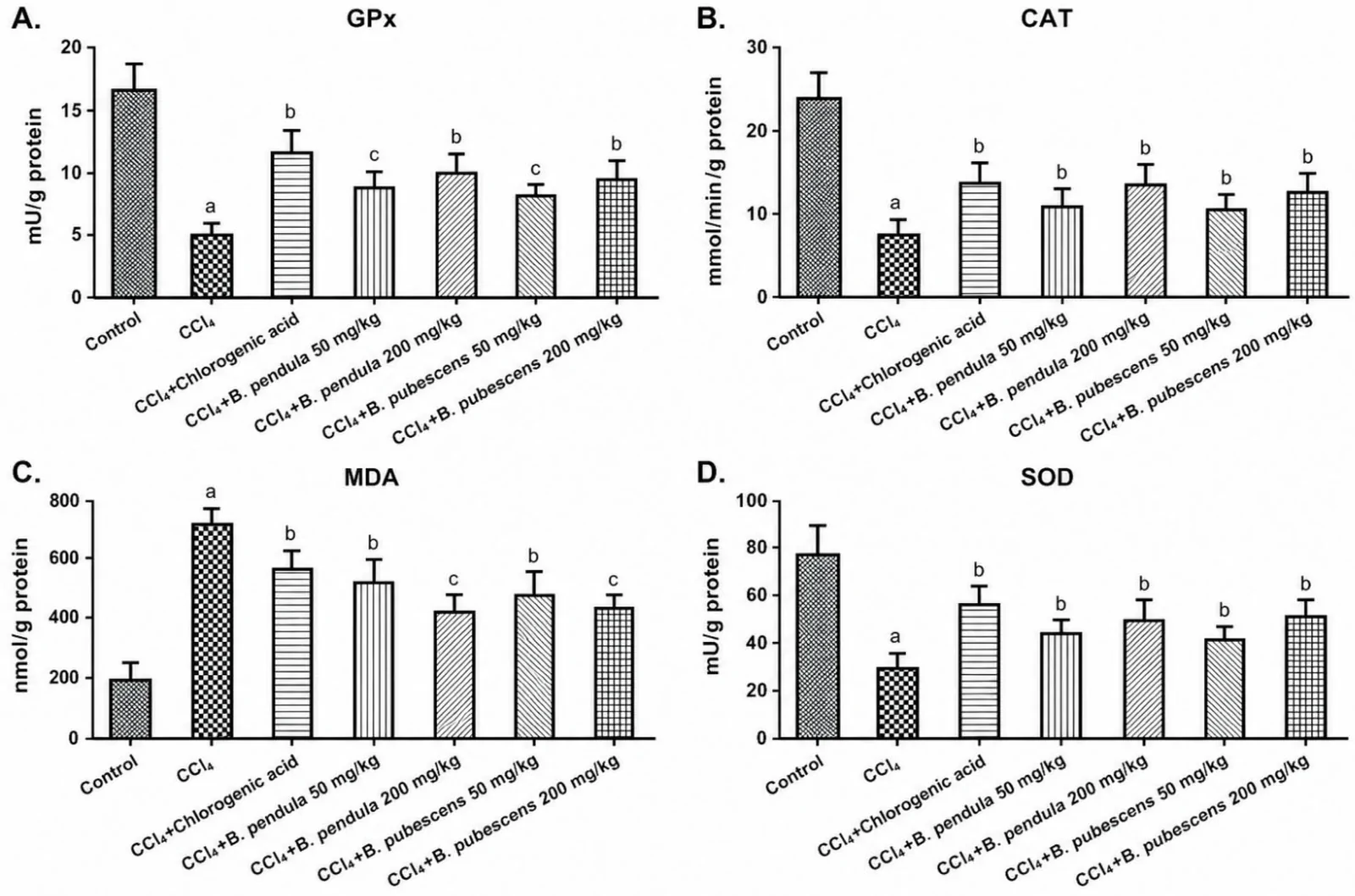
Effects of chlorogenic acid and *Betula* spp. extracts on: GPx (**A**); CAT (**B**); MDA (**C**); and SOD (**D**) (mean ± SD, 5 animals/group). a—*p* < 0.05 compared to the control group; b—*p* < 0.05 compared to the CCl_4_ group; c—*p* < 0.05 compared to the CCl_4_ + chlorogenic acid group; between different doses of the same extract.

The effects of chlorogenic acid and *Betula* spp. extracts on oxidative stress parameters in liver tissue are presented in Figure 4. Administration of CCl_4_ resulted in a marked imbalance in the antioxidant defense system, characterized by significantly decreased GPx, CAT, and SOD activities and increased MDA levels compared with the control group (*p* < 0.0001 for all parameters). Treatment with chlorogenic acid and *Betula* spp. extracts significantly improved antioxidant status compared with the CCl_4_ group. GPx activity was significantly increased by chlorogenic acid (*p* < 0.0001), *B. pendula* at 50 mg/kg (*p* < 0.01) and 200 mg/kg (*p* < 0.0001), and *B. pubescens* at 50 mg/kg (*p* < 0.01) and 200 mg/kg (*p* < 0.0001). CAT activity was significantly increased by chlorogenic acid (*p* < 0.0001), *B. pendula* at 50 mg/kg (*p* < 0.05) and 200 mg/kg (*p* < 0.0001), and *B. pubescens* at 50 mg/kg (*p* < 0.05) and 200 mg/kg (*p* < 0.001). MDA levels were significantly reduced by chlorogenic acid and by both doses of both *Betula* spp. extracts (*p* < 0.0001 for all comparisons versus CCl_4_). SOD activity was significantly increased by chlorogenic acid (*p* < 0.0001), *B. pendula* at 50 mg/kg (*p* < 0.05) and 200 mg/kg (*p* < 0.001), and *B. pubescens* at 50 mg/kg (*p* < 0.05) and 200 mg/kg (*p* < 0.0001). These findings indicate attenuation of CCl_4_-induced oxidative stress by both *Betula* spp. extracts, although the magnitude of the effect varied between doses and parameters.

Among the tested compounds, chlorogenic acid exhibited a pronounced effect on the restoration of antioxidant enzyme activity, while *Betula* spp. extracts also demonstrated significant antioxidant effects, although with some variability between doses and parameters. In general, higher doses (200 mg/kg) tend to produce a more marked improvement compared to lower doses (50 mg/kg), particularly in the case of GPx and SOD activity, as well as MDA reduction.

Overall, the observed changes indicate that *Betula* spp. extracts significantly counteract CCl_4_-induced oxidative stress, contributing to the restoration of the hepatic antioxidant defense system and the reduction in lipid peroxidation.

Upon macroscopic examination, the animals in the toxic control group presented with mild icterus (jaundiced appearance) and slight hepatomegaly, whereas splenomegaly was not observed. A small volume of free peritoneal fluid (ascites) was detected in only a single animal. Overall, despite the induced hepatic injury, the cohort exhibited a relatively preserved general body scoring condition without signs of severe systemic distress. These alterations were less visible in the groups benefiting from therapy, in which only some mild hepatomegaly was seldomly observed in some individuals.

**Figure 5:**
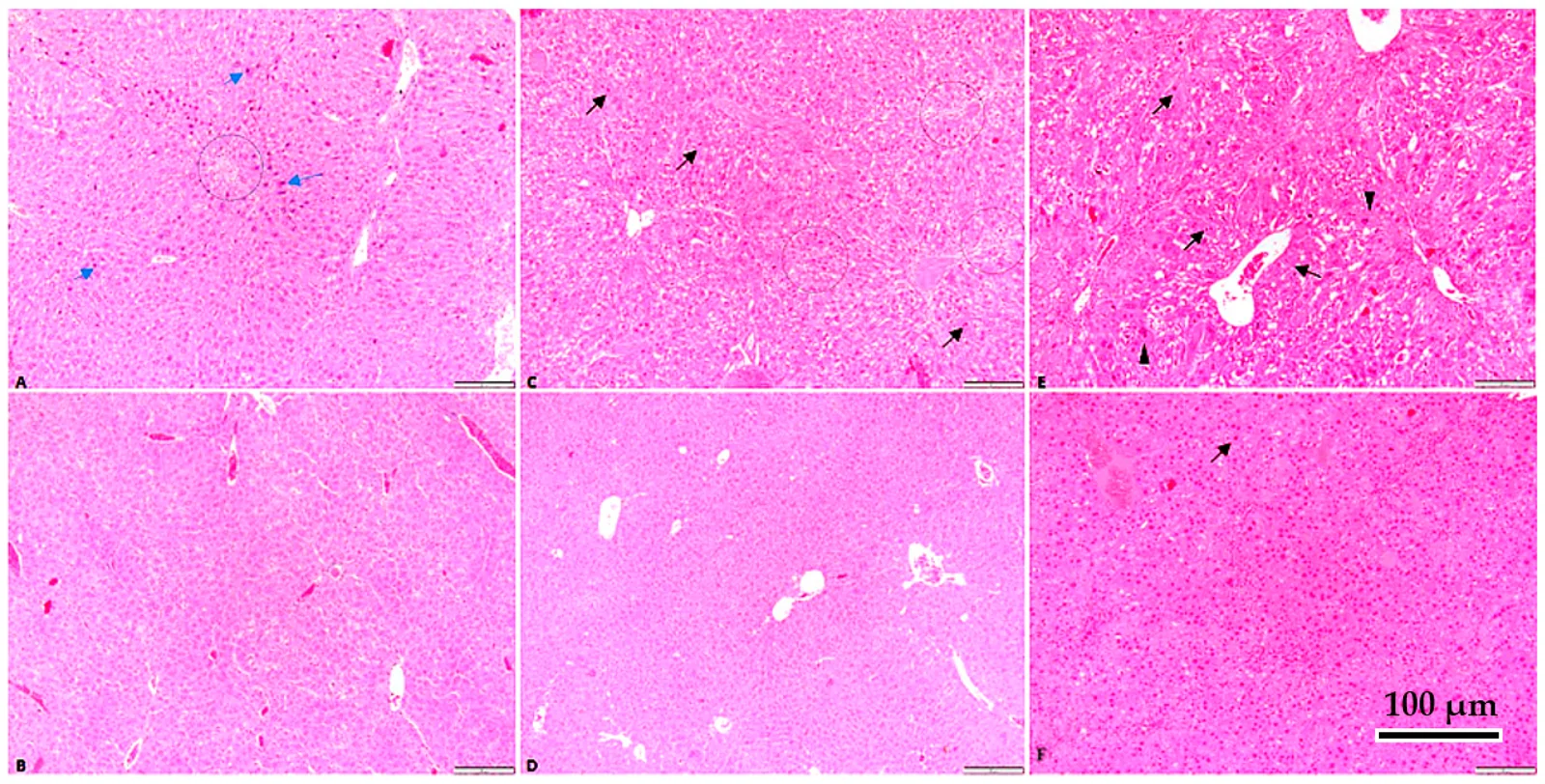
Photomicrograph of the hepatic lesions: (**A**) individual cell necrosis (arrows); minimal centrolobular infiltration predominated by macrophages (circled area); (**B**) minimal diffuse vacuolar degeneration; (**C**) numerous mitotic figures (arrows); multifocal mononuclear inflammatory infiltrate (circled areas); diffuse vacuolar degeneration; (**D**) diffuse minimal vacuolar degeneration; (**E**) diffuse necrosis of hepatocytes, accompanied by inflammatory infiltrates, numerous mitotic figures (arrows) and presence of multinucleated cells (arrowhead); (**F**) moderate anisokaryosis, presence of few mitoses (arrow) and centrilobular inflammatory infiltrates. Groups: (**A**) CCl_4_; (**B**) CCl_4_ + chlorogenic acid; (**C**) CCl_4_ + *B. pendula* extract 50 mg/kg; (**D**) CCl_4_ + *B. pendula* extract 200 mg/kg; (**E**) CCl_4_ + *B. pubescens* extract 50 mg/kg; (**F**) CCl_4_ + *B. pubescens* extract 200 mg/kg.

Histopathological examination of liver sections revealed distinct alterations between experimental groups (Figure 5). The control group exhibited normal hepatic architecture, with preserved lobular organization and no evident morphological changes in the majority of individuals.

In the CCl_4_-treated group, hepatic lesions were characterized by minimal centrolobular and moderate periportal inflammatory infiltrate, composed predominantly of macrophages and occasional neutrophils. Hepatocellular injury was evident, with medio- and centrolobular necrosis, characterized by hepatocytes with hypereosinophilic cytoplasm and pyknotic nuclei. Numerous hepatocytes exhibited karyomegaly, indicating cellular stress and regenerative response.

In the chlorogenic-acid-treated group, the hepatic histoarchitecture was only mildly altered, with lobular organization less clearly defined. Minimal diffuse vacuolar degeneration was observed, along with an increased number of Kupffer cells, suggesting activation of resident macrophages, potentially associated with regenerative processes.

In the *B. pendula* 50 mg/kg group, pronounced hepatic alterations were observed, including multifocal hepatocellular necrosis, diffuse congestion, and microvesicular steatosis. Numerous hepatocytes displayed multinucleation and karyomegaly, along with frequent mitotic figures, some of which appeared atypical. A multifocal mononuclear inflammatory infiltrate was also present.

In contrast, the *B. pendula* 200 mg/kg group exhibited markedly reduced lesions. Hepatocellular vacuolar degeneration was present but moderate and diffuse, while inflammatory infiltrate and necrotic foci were absent. In addition, a granular brown–yellow pigment, suggestive of ceroid or bile pigment accumulation, was observed within hepatocyte cytoplasm.

The *B. pubescens* 50 mg/kg group showed severe hepatic alterations, with diffuse degenerative and inflammatory changes. Numerous necrotic foci were identified, accompanied by loss of normal histoarchitecture and hepatocytes in various stages of necrosis, including karyolysis, karyorrhexis, and cytolysis. Hepatocytes frequently exhibited pale, vacuolated cytoplasm, consistent with steatosis or vacuolar degeneration. Increased mitotic activity, including atypical figures, as well as binucleated and multinucleated cells, karyomegaly, and cytomegaly were evident. A multifocal inflammatory infiltrate, predominantly composed of polymorphonuclear cells, was observed, occasionally associated with mild fibrosis and congestion.

In the *B. pubescens* 200 mg/kg group, lesions were similar in nature to those observed in the 50 mg/kg group but of reduced intensity. Hepatocellular necrosis and vacuolar degeneration are still present, along with anisocytosis and anisokaryosis. Mitotic figures, including atypical forms, were observed, and inflammatory infiltrate was predominantly localized in the centrolobular region.

## 3. Discussion

### 3.1. Phytochemistry and Antioxidant Activity

Understanding the foliar polyphenolics of *B. pendula* and *B. pubescens* is essential to their roles in plant defense, herbivore interactions, UV protection, and chemistry. The results obtained for *B. pendula* leaf extract compared to the literature data are generally consistent with those reported in previously performed studies [10,14,22,23,24,25]. Thus, Costea et al. reported lower concentrations of polyphenols, CADC and flavonoids in the dried leaves of *B. pendula* [22], while Penkov et al. found higher flavonoid levels [14]. At the same time, Sevastre-Berghian et al. determined a lower phenolic content and a lower antioxidant activity, evaluated by the DPPH and FRAP methods [23]. The only study reporting a higher polyphenolic total, but a lower antioxidant capacity is the one performed by Kucharski et al. [25]. Differences can be explained by variations in the processing of plant material, together with harvesting and pedoclimatic conditions, but also of extraction methods and solvent polarity. Regarding the published data on the polyphenol content of *B. pubescens* leaves, they are very few. In the present study, the *B. pendula* leaf extract showed higher antioxidant activity than *B. pubescens* leaves, in relation to a higher content of total polyphenols, while *B. pubescens* is a better source of antioxidant flavonoids.

The results of the LC/MS/ESI analysis confirm that quercetin derivatives and hydroxycinnamic acids represent the dominant classes of phenolic compounds in *Betula* spp. leaves, which is consistent with previous phytochemical investigations of the genus [1,5,11,12,13,15,22,25,26,27].

Among the identified compounds, isoquercitrin was the most abundant flavonoid in *B. pendula* leaves (28.68 ± 3.82 µg/mL), while quercitrin reached the highest concentration in *B. pubescens* (29.83 ± 0.84 µg/mL). Previous observations have been reported in earlier studies describing birch leaf phytochemistry, where quercetin glycosides, particularly isoquercitrin, hyperoside and quercitrin, were identified as major phenolic constituents responsible for many of the biological properties of *Betula* spp. extracts [12,15,22,27]. However, although quercitrin (quercetin-3-O-rhamnoside) has previously been reported in *B. pubescens*, the available literature does not consistently identify it as the predominant flavonoid in this species. For instance, Raal et al. reported quercitrin among the major flavonoids of *B. pubescens* leaves, but hyperoside/isoquercitrin occurred at considerably higher concentrations and was described as the principal flavonoid component [27]. Therefore, the particularly high quercitrin level observed in *B. pubescens* in the present study represents a distinctive quantitative feature of the investigated samples rather than a consistently reported chemotaxonomic characteristic of the species. This discrepancy may reflect the well-recognized variability of plant secondary metabolism in response to geographical origin, genotype, environmental conditions, phenological stage, and harvesting period, as well as differences in extraction and analytical methodologies. Thus, our findings confirm previous reports regarding the occurrence of quercitrin in *B. pubescens*, while also highlighting substantial quantitative variability in the flavonoid profile of this species. In agreement with the phytochemical profile observed for *B. pendula* in the present study, investigations performed by Keinänen et al. demonstrated that *Betula pendula* leaves contain significant amounts of quercetin derivatives, including hyperoside and isoquercitrin, which contribute to the antioxidant and anti-inflammatory activities [26]. An important flavonoid detected in both species was hyperoside, with higher levels in *B. pubescens* (15.07 ± 0.06 µg/mL) compared with *B. pendula* (10.36 ± 0.20 µg/mL). Hyperoside has been widely reported as one of the characteristic flavonol glycosides in *Betula* spp. leaves and is known to possess antioxidant, hepatoprotective, and anti-inflammatory activities [4,22,28]. Previous studies on *B. pendula* leaf extracts have shown that hyperoside can significantly contribute to the pharmacological properties traditionally attributed to birch preparations [12,29].

In our study, the phenolic acid fraction was dominated by cryptochlorogenic acid, which was present in relatively high amounts in both species, with slightly higher concentrations in *B. pubescens*. Chlorogenic acid and its isomers, including neochlorogenic and cryptochlorogenic acids, are commonly reported in *Betula* spp. leaves and have been associated with antioxidant and hepatoprotective activities [27,29]. Similar findings have been reported for *B. pubescens*, where hydroxycinnamic acids represented a significant portion of the phenolic profile [27]. Gallic acid was also detected in both extracts, with slightly higher concentrations in *B. pubescens*. This compound has been previously reported in *Betula* species and is known for its strong antioxidant and antimicrobial properties. In addition, ferulic acid and caffeic acid, although present in lower concentrations, are typical phenolic acids identified in many medicinal plants and contribute to their biological activity through radical-scavenging and anti-inflammatory mechanisms [1,12]. Interestingly, several flavonoids exhibited species-dependent distribution patterns. For instance, tilianin was significantly more abundant in *B. pendula*, whereas naringenin and apigenin were present in higher amounts in *B. pubescens*. This is the first study, to the best of our knowledge, to report the presence of tilianin in the composition of both species. Such variations in secondary metabolite composition between closely related *Betula* species have been previously reported and are often attributed to genetic factors, environmental conditions, geographical origin, and seasonal variations affecting biosynthetic pathways [11,27,30,31]. Another noteworthy observation concerns *trans-p*-coumaric acid, which was detected only in *B. pendula* leaves, while its concentration in *B. pubescens* was below the quantification limit. Differences in phenolic acid distribution between birch species have also been described in previous studies [10] and may reflect species-specific metabolic regulation of the phenylpropanoid pathway.

Overall, the polyphenolic profile obtained in the present study is consistent with previously published phytochemical data for *Betula* spp. leaves, which typically highlight the predominance of flavonol glycosides derived from quercetin and kaempferol, along with hydroxycinnamic acid derivatives. However, the quantitative differences observed between *B. pendula* and *B. pubescens* suggest that each species possesses a distinct metabolic fingerprint that may influence their pharmacological potential.

### 3.2. Cytotoxicity

The effects of hydroalcoholic leaf extracts obtained from *B. pubescens* (S5) and *B. pendula* (S6) were evaluated in LX-2 cells, an immortalized human hepatic stellate cell line, and HepG2 cells, a human hepatocellular carcinoma cell line widely used in studies of hepatic metabolism and liver cancer. Cell viability was expressed as a percentage relative to untreated control cells, which was considered 100%. In LX-2 cells, treatment with both extracts resulted in viability values close to or slightly above the control level, indicating that neither extract substantially reduced cell viability under the experimental conditions. The *B. pubescens* extract (S5) produced mean viability values ranging from approximately 95% to 103%, whereas the *B. pendula* extract (S6) showed mean viability values ranging from approximately 98% to 106%. In several cases, viability values exceeded the control level, which may reflect variations in cellular metabolic activity rather than a specific stimulatory or protective effect. Overall, both extracts maintained high LX-2 cell viability across the tested concentration ranges. In contrast, a more pronounced reduction in cell viability was observed in HepG2 cells following treatment with the *B. pubescens* extract (S5), with mean viability values ranging from approximately 71% to 78%. The *B. pendula* extract (S6) produced higher mean viability values, ranging from approximately 85% to 100%, indicating a less pronounced reduction in HepG2 cell viability under the tested conditions. Thus, *B. pubescens* induced a greater reduction in HepG2 cell viability than *B. pendula*. However, none of the tested concentrations resulted in a 50% reduction in cell viability, and therefore an experimental IC_50_ could not be determined within the investigated concentration range. The greater reduction in HepG2 cell viability observed following treatment with *B. pubescens* may be related to differences in the phytochemical composition of the two extracts, including their phenolic profiles. Phenolic compounds have previously been associated with modulation of cellular redox status and signaling pathways involved in cell survival. However, these mechanisms were not directly investigated in the present study and therefore remain speculative. The observed differences should consequently be interpreted as extract- and cell-line-dependent changes in cell viability rather than as evidence of a specific cytotoxic or molecular mechanism. Further studies using complementary cellular and mechanistic assays are required to better understand the mechanisms underlying these effects. The present study also has some limitations that should be considered when interpreting the results. The biological effects of the extracts were evaluated under specific experimental conditions and using selected cell models, which may not fully reflect their effects in other cellular systems or more complex biological settings. In addition, the CCK-8 assay provides information on cell viability and metabolic activity, but further complementary approaches would be needed to better characterize the biological mechanisms involved. The findings should therefore be considered preliminary and provide a basis for further investigations using additional cell models, assays, and experimental conditions.

Evidence from multiple studies indicates that leaves and buds of *B. pendula* and *B. pubescens* are rich in bioactive constituents, predominantly flavonoids (including aglycone forms) and triterpenoids, which demonstrate cytotoxic and antiproliferative properties against various human cancer cell lines under in vitro conditions. Aglycone flavonoids, notably santin and cirsimaritin, trigger apoptosis via both intrinsic and extrinsic pathways, thereby reducing cell viability in gastric, colorectal, and hepatic cancer models; glycosylated flavonoid derivatives generally exhibit diminished cytotoxic potency relative to their aglycone counterparts, a pattern consistent with the broader pharmacology of flavonoids [9]. Studies performed by Isidorov et al. further elaborate that triterpenes and flavonoids isolated from *Betula* spp. are responsible for cytotoxic activity, with leaf-derived activity attributable primarily to aglycone flavonoids and other lipophilic constituents; their findings suggest that leaf extracts can be both cytotoxic and antiproliferative, while in some cases displaying comparatively lower toxicity toward certain normal cell types, contingent on the specific extract and cell line examined [32]. Several other important observations derive from bud or combined bud-leaf preparations; leaf-specific cytotoxicity data, where they exist, are frequently inferred from bud-based studies or from crude leaf extracts evaluated in cytotoxicity assays. Consequently, the precise quantitative potency of leaf extracts in isolation—as distinct from bud extracts or chaga harvested from birch—remains insufficiently and inconsistently characterized across the published literature, necessitating cautious interpretation when extending bud-derived cytotoxic mechanisms to leaf-based material. Some leaf- and bud-derived extracts have been shown to exert cytotoxic effects on normal fibroblasts, pointing to potential toxicity toward non-cancerous cells; conversely, other investigations suggest differential sensitivity between normal and malignant cells depending on extract composition and concentration. This variability underscores the necessity for standardized comparative testing panels incorporating both cancerous and non-cancerous cell lines, alongside uniform extraction methodologies, to reliably assess selectivity [9,32]. Additionally, Efthimiou et al. report that certain *B. pendula* leaf-derived products display cytotoxic activity accompanied by genotoxic effects in vitro; such findings warrant rigorous evaluation of therapeutic windows and safety profiles within any translational research framework [33].

### 3.3. In Vivo Hepatoprotection and Histopathology

The present study evaluated the effects of chlorogenic acid and *Betula* spp. extracts on biochemical parameters in a CCl_4_-induced model of hepatotoxicity, a well-established model characterized by oxidative stress, lipid peroxidation, and hepatocellular damage. The hepatotoxicity of CCl_4_ is primarily driven by its metabolic bioactivation via hepatic CYP2E1, which yields highly reactive trichloromethyl free radicals. These radicals initiate a cascade of lipid peroxidation, reflected in increased MDA levels, down-regulation in antioxidant enzyme activity (GPx, CAT, and SOD). A main target or oxidative stress is hepatocyte membrane integrity, leading to cellular necrosis. Therefore, administration of CCl_4_ led to significant alterations in serum biochemical markers, including elevated transaminase activity (ALT and AST). The subsequent release of damage-associated molecular patterns (DAMPs) activates resident Kupffer cells through the NF-kappa B pathway. This activation triggers an inflammatory response characterized by the upregulation of pro-inflammatory cytokines, such as TNF-alpha, IL-1, and IL-6. In response, the liver aggressively upregulates positive acute-phase proteins (like C-Reactive Protein and Serum Amyloid A) rising the plasma globulin levels. Concurrently, negative acute-phase proteins (like albumin) drop significantly as the liver diverts its amino acid resources toward defense and structural repair. Furthermore, the inflammatory cytokines locally sustain chronic hepatic tissue injury and drive subsequent fibrogenesis. Cholestasis adjacent to liver inflammation elevated ALP activities, as well as serum bilirubin and bile acids levels, while hepatocellular damage was also evident [34]. Chronic liver injury was induced via oral gavage of CCl_4_ at a dose of 1.0 mL/kg body weight, administered three times per week for six consecutive weeks. The orogastric route was selected over intraperitoneal injection to utilize hepatic first-pass metabolism via the portal system, and to completely prevent the confounding risks of chemical peritonitis and intra-abdominal adhesions common to long-term chronic studies. Notably, this optimized intragastric protocol achieved a 100% survival rate across all cohorts, ensuring no animal loss or attrition bias prior to the final analysis.

Body weight changes represent a general indicator of systemic toxicity and overall metabolic status in experimental models of liver injury [35]. Interestingly, no significant differences in total body weight were observed between the toxic control and normal control group over the 6-week period, the body weight been relatively preserved during the study. This finding, in conjunction with a relatively preserved body scoring condition and only slight hepatomegaly, and the absence of splenomegaly or significant ascites, suggests that the regimen successfully localized advanced hepatic injury without triggering severe systemic cachexia or animal attrition, balancing high experimental yield with improved animal welfare.

Treatment with chlorogenic acid and *Betula* spp. extracts resulted in a general attenuation hepatocellular damage, as evidenced by the reduction in ALT and AST activities compared to the toxic control group. Although the degree of improvement varies between groups and parameters, this effect suggests a protective influence on hepatocellular integrity. The observed decrease in transaminase levels may be associated with the ability of the tested compounds to stabilize cellular membranes and reduce enzyme leakage, a mechanism frequently reported for plant-derived antioxidants and polyphenolic compounds [36]. Alterations in protein metabolism induced by CCl_4_, particularly the decrease in albumin levels and the changes in total protein and globulin concentrations, further indicate impaired synthetic liver function. The partial normalization of these parameters following treatment suggests an improvement in hepatic biosynthetic capacity. This effect may be attributed to the bioactive constituents of the extracts, which are known to exert anti-inflammatory and antioxidant actions, thereby supporting the restoration of normal hepatic function [37]. Elevated activity of ALP and high serum levels of total bilirubin and total bile acids in the CCl_4_ group reflect disturbances in hepatic excretory function and bile flow, which are commonly associated with toxic hepatitis. The alleviation of these parameters in treated groups indicates a potential improvement in bile metabolism and excretory processes. Such effects have been previously associated with phenolic compounds, including chlorogenic acid, which have been shown to modulate oxidative stress and improve hepatic function in experimental models [38]. Overall, the effects observed in this study suggest that both chlorogenic acid and *Betula* spp. extracts exert a hepatoprotective influence, likely mediated through antioxidant and membrane-stabilizing mechanisms. The slightly more pronounced effects observed at higher doses may indicate a dose-related response.

The present study demonstrated that CCl_4_ administration induces a pronounced state of oxidative stress in hepatic tissue, as reflected by the significant decrease in antioxidant enzyme activities (GPx, CAT, and SOD) and the concomitant increase in lipid peroxidation, evidenced by elevated MDA levels. Treatment with chlorogenic acid and *Betula* spp. extracts resulted in a significant restoration of antioxidant enzyme activity and a reduction in MDA levels compared to the CCl_4_ group, indicating attenuation of oxidative stress. The increase in GPx, CAT, and SOD activities suggests a recovery of the enzymatic antioxidant defense system, which plays a critical role in neutralizing reactive oxygen species and maintaining cellular redox balance [39]. In parallel, the reduction in MDA levels reflects a decrease in lipid peroxidation, suggesting protection of membrane integrity and reduced oxidative damage. The observed antioxidant effects may be attributed to the presence of phenolic compounds, particularly chlorogenic acid, which is known for its strong radical-scavenging properties and its ability to modulate oxidative-stress-related pathways [40]. In addition, *Betula* species are rich in bioactive constituents, including flavonoids and triterpenes, which have been reported to exert antioxidant and hepatoprotective effects through multiple mechanisms, including enhancement of endogenous antioxidant defenses and inhibition of lipid peroxidation [25]. Although both chlorogenic acid and *Betula* spp. extracts demonstrated significant antioxidant activity, some variability was observed between doses and parameters, suggesting that the response may depend on the specific compound composition and the interaction between different bioactive constituents. The slightly more pronounced effects observed at higher doses support a dose-related response; however, the lack of consistency across all markers indicates that the relationship between dose and antioxidant efficacy is not strictly linear. Overall, the results indicate that the tested compounds effectively mitigate CCl_4_-induced oxidative stress, contributing to the restoration of hepatic redox homeostasis. Given the central role of oxidative stress in the pathogenesis of liver injury, these findings provide mechanistic support for the hepatoprotective effects observed in the present study and highlight the potential of chlorogenic acid and *Betula* spp. extracts as therapeutic agents targeting oxidative-stress-mediated hepatic damage.

Histopathological evaluation confirmed the hepatic damage induced by CCl_4_ and provided structural evidence supporting the biochemical and oxidative stress findings. The lesions observed in the CCl_4_ group, including centrolobular necrosis, inflammatory infiltrate, and hepatocellular alterations such as karyomegaly and cytoplasmic hypereosinophilia, are characteristic of CCl_4_-induced hepatotoxicity and reflect the well-documented mechanism involving the formation of reactive metabolites and subsequent lipid peroxidation. These processes lead to membrane damage, loss of cellular integrity, and activation of inflammatory pathways, ultimately resulting in hepatocellular necrosis and architectural disruption [41]. Treatment with chlorogenic acid resulted in a clear attenuation of these lesions, as evidenced by the preservation of hepatic structure, minimal vacuolar degeneration, and the absence of significant necrotic or inflammatory changes. The increase in Kupffer cell number observed in this group may indicate activation of hepatic macrophages involved in the clearance of cellular debris and in the promotion of tissue repair, suggesting a regenerative component associated with the hepatoprotective effect. These findings are consistent with the known antioxidant and anti-inflammatory properties of chlorogenic acid, which contribute to the reduction in oxidative stress and stabilization of cellular membranes [42]. The effects of *B. pendula* were dose-dependent and highlighted a differential response between the two tested concentrations. At 50 mg/kg, the presence of multifocal necrosis, inflammatory infiltrate, and steatosis indicates that this dose was insufficient to counteract CCl_4_-induced damage. In contrast, the 200 mg/kg dose markedly reduces the severity of lesions, with the absence of necrosis and inflammatory infiltrate, suggesting a protective effect at higher concentrations. The presence of intracellular pigment, likely ceroid or bile pigment, may reflect ongoing metabolic processing or residual oxidative damage, but in the absence of active necrosis or inflammation, it does not appear to be associated with severe injury. In the case of *B. pubescens*, the histopathological findings indicate a less pronounced protective effect compared to *B. pendula*. The 50 mg/kg dose was associated with extensive hepatic damage, including widespread necrosis, inflammatory infiltration, and loss of normal architecture, suggesting limited efficacy at this concentration. Although the 200 mg/kg dose showed a reduction in lesion severity, degenerative and necrotic changes were still present, indicating only partial protection. The persistence of anisocytosis, anisokaryosis, and atypical mitotic figures suggests ongoing cellular stress and regeneration, rather than complete recovery. Overall, the histopathological findings agree with the biochemical and oxidative stress results, supporting the conclusion that chlorogenic acid and *B. pendula*, particularly at higher doses, exert hepatoprotective effects, likely mediated through antioxidant mechanisms and the preservation of cellular integrity. In contrast, *B. pubescens* demonstrated a weaker and less consistent protective profile, indicating possible differences in phytochemical composition and biological activity between the two species.

Some differences were observed between the magnitude of biochemical improvement and the histopathological findings. This apparent discrepancy may reflect the different biological information provided by these endpoints. Serum biochemical and oxidative-stress markers reflect functional hepatocellular injury and redox status at the time of sampling, whereas histopathological alterations represent accumulated structural damage and may persist despite partial functional recovery. Accordingly, the persistence of necrotic, degenerative, or inflammatory lesions in some treatment groups, particularly at 50 mg/kg, suggests that biochemical improvement should not be interpreted as complete morphological recovery. The greater concordance between biochemical improvement and reduced lesion severity at 200 mg/kg, particularly for *B. pendula*, further supports a dose-dependent but incomplete hepatoprotective effect.

The comparison between phytochemical composition and biological activity suggests that the stronger hepatoprotective effect of *B. pendula* may be related to its higher total phenolic content, higher chlorogenic acid concentration, and greater antioxidant capacity. However, *B. pubescens* contained higher amounts of several individual flavonoids, including hyperoside and quercitrin, despite showing a less pronounced protective effect. These findings indicate that hepatoprotective activity is likely determined by the overall phytochemical profile and the combined effects of multiple constituents rather than by a single metabolite.

## 4. Materials and Methods

### 4.1. Chemicals and Reagents

All chemicals and solvents used in this study were of analytical grade and obtained from various commercial suppliers. Aluminum chloride, Arnow reagent, Folin–Ciocâlteu reagent, ethanol, methanol, sodium acetate, hydrochloric acid, acetic acid, DPPH, acetonitrile, and ammonium acetate were purchased from Merck KGaA (Darmstadt, Germany). Ferric chloride (III), sodium hydroxide, and sodium carbonate were supplied by Alfa Aesar (Ward Hill, MA, USA/Karlsruhe, Germany). Trolox (6-hydroxy-2,5,7,8-tetramethylchroman-2-carboxylic acid) and 2,4,6-tripyridyl-s-triazine (TPTZ) were obtained from Sigma-Aldrich (Steinheim, Germany). Reference standards used for LC/MS/ESI analysis were acquired from PhytoLab (Vestenbergsgreuth, Germany). Spectrophotometric measurements were carried out using an Agilent Technologies Cary 60 UV–Vis spectrophotometer (Santa Clara, CA, USA). Reagents required for cytotoxicity testing were obtained from the following sources: the LX-2 (CVCL_5792) and HepG2 cell lines were generously provided by Medfuture, Cluj-Napoca, Romania; Dulbecco’s Modified Eagle Medium (DMEM) was supplied by Gibco (Paisley, UK); fetal bovine serum, L-glutamine, and antibiotic–antimycotic solution were obtained from Sigma-Aldrich (St. Louis, MO, USA).

### 4.2. Preparation of Extracts

The vegetal material was harvested from the ecological culture of PlantExtrakt (Rădaia, Cluj County, Romania), in September 2024. Vegetal material was identified by Lecturer Irina Ielciu, PhD and voucher specimens were deposited in the herbarium of the Pharmacognosy Department of the Faculty of Pharmacy Cluj-Napoca (Voucher no. 962). The *Betula* spp. tinctures were prepared according to the German Homeopathic Pharmacopeia (GHPh) and European Pharmacopeia (EPh) specifications for tincture preparation (method 1.1.5). Fresh leaves were extracted by cold maceration with 90% *v*/*v* ethanol. One part of the crushed vegetal material was mixed with 3 parts of ethanol for *B. pendula* and with 5 parts of ethanol for *B. pubescens* and shaken 2 times per day for 10 days. Afterwards, the extracts were filtered. For the physical characterization of the obtained tinctures, relative density, dry residue at evaporation and ethanol content were assessed according to the methods of the European Pharmacopeia. Before using the animal experiment, the tinctures were evaporated maintaining temperature below 40 °C under vacuum, until the complete removal of alcohol and immediately administered to animals. The relative density was assessed according to the European Pharmacopeia (EPh) using a Mettler Toledo (Greifensee, Switzerland) digital densimeter. The dry residue was evaluated according to the European Pharmacopeia (EPh) using a Kern analytical scale (Berlin, Germany) and a Memmert drying cabinet (Schwabach, Germany). The ethanol content was assessed according to European Pharmacopeia (EPh) using a Neo-Clevenger apparatus (LaborXing, Shenzhen, China) and a Mettler Toledo digital densimeter [23,43]. The physical properties of the extract were evaluated for the relative density at 0.983 g/cm^3^ for *B. pubescens* and at 0.979 g/cm^3^ for *B. pendula,* while for the dry residue the values were at 3.95% for *B. pubescens* and at 4.47% for *B. pendula*. The ethanolic content was established at 28% *v*/*v* for *B. pubescens* and at 33% for *B. pendula*.

### 4.3. Determination of Total Phenolic Content (TPC) by the Folin–Ciocâlteu Method

The total polyphenol content (TPC) of the two *Betula* spp. leaf extracts (*B. pendula* and *B. pubescens*) was determined using the Folin–Ciocâlteu spectrophotocolorimetric method. Briefly, the extracts were mixed with the Folin–Ciocâlteu reagent, followed by the addition of a 29% sodium carbonate solution. The reaction mixtures were incubated under appropriate conditions to allow for color development (blue), after which the absorbance was measured at 760 nm. TPC was quantified using a calibration curve constructed with gallic acid as standard (R^2^ = 0.998). The results were expressed in mg gallic acid equivalents (GAE)/g of dry weight (dw) plant material [44,45,46,47].

### 4.4. Spectrophotometric Determination of Total Flavonoid Content (TFC)

The total flavonoid content (TFC) of *Betula* spp. extracts was determined using the aluminum chloride spectrophotometric. Briefly, the extracts were reacted with a 25% aluminum chloride solution to form a yellow compound flavonoid–aluminum complex. The absorbance of the resulting complex was measured at 430 nm. Quantification was performed using a rutin calibration curve (R^2^ = 0.998), and the results obtained as the average of three independent determinations were expressed in mg of rutin equivalents (ER)/g of dry weight (dw) plant material [44,45,46,47].

### 4.5. Spectrophotometric Determination of Caffeic Acid Derivative (CADC)

The content of caffeic acid derivatives (CADC) in *B. pendula* and *B. pubescens* fresh leaf extracts was spectrophotometrically determined using Arnow reagent, a mixture of sodium nitrite (NaNO_2_) and sodium molybdate (Na_2_MoO_4_). Absorbance was measured at 500 nm, and quantification was performed using a calibration curve prepared with caffeic acid as the phenolic acid standard (R^2^ = 0.989). Results were expressed in mg caffeic acid equivalents (CAE)/g of dry weight (dw) plant material [44,45,46,47].

### 4.6. In Vitro Antioxidant Activity Assays

#### 4.6.1. DPPH Assay

The antioxidant activity of the two *Betula* spp. extracts was evaluated using the DPPH radical scavenging assay. This method is based on the ability of natural polyphenols to donate an electron or a hydrogen atom to the DPPH• free radical, resulting in its neutralization from a purple to a light-yellow compound. The decrease in absorbance was monitored spectrophotometrically at 517 nm, and the antioxidant activity of the samples was expressed as IC_50_ (mg/mL), defined as the extract concentration required to scavenge 50% of the DPPH• radicals, compared to Trolox. All measurements were performed in triplicate, and lower IC_50_ values indicated stronger antioxidant activity [44,45,46,47].

#### 4.6.2. FRAP Assay

The antioxidant capacity of *B. pendula* and *B. pubescens* extracts was also evaluated using the FRAP assay. This method is based on the ability of antioxidant compounds present in the two samples to reduce ferric ions to ferrous ions, resulting in the formation of a blue Fe^2+^–TPTZ complex. The absorbance of the reaction mixture was measured spectrophotometrically at 450 nm, and quantification was performed using a calibration curve prepared with Trolox as the reference antioxidant (R^2^ = 0.998). The results were expressed in microM Trolox equivalents (µM TE)/mL of extract [44,45,46,47].

### 4.7. LC/MS/ESI Analysis

Identification and quantification of polyphenolic compounds were performed using an LC/MS/ESI system (Shimadzu Nexera I coupled with an LC/MS-8045 triple quadrupole mass spectrometer, Kyoto, Japan) equipped with a quaternary solvent delivery unit, autosampler, electrospray ionization (ESI) interface, and quadrupole analyzer. Chromatographic separation was carried out on a reverse-phase Luna C18 column (150 × 4.6 mm, 3 µm particle size, 100 Å pore size; Phenomenex, Torrance, CA, USA), with the column oven maintained at 40 °C. The mobile phase consisted of methanol and ultrapure water obtained from a Simplicity water purification system (Merck Millipore, Billerica, MA, USA), both acidified with 0.1% formic acid. Elution was performed using a gradient program (Table 4) at a constant flow rate of 0.5 mL/min, with a total run time of 36 min. A panel of reference standards was used for compound identification, including phenolic acids flavonoids, and related phenolic derivatives such as *trans-p* coumaric acid, salicylic acid, chlorogenic acid, neochlorogenic acid, crytochlorogenic acid, caffeic acid, ferulic acid, gallic acid, synapic acid rosmarinic acid; kaempferol, naringenin, myricetin, apigenin, quercetin, acacetin, chrysin, diosmin, hesperetin and its derivatives; tilianin, isoquercitrin, quercitrin, luteolin-7*-O*-glucosid, hyperoside, rutin, vitexin, and other structurally related polyphenols. Mass spectrometric detection was performed using an electrospray ionization source operating in both positive and negative ionization modes. Data acquisition was carried out in multiple reaction monitoring (MRM) mode. Detailed MRM transitions and optimized parameters are presented in Table 1. For the vaporization step, nitrogen was used as the drying gas at 30 psi with a flow rate of 10 L/min. The capillary voltage was maintained at +3000 V. Injection volumes were set to 1 µL for reference standards and 10 µL for sample extracts. Compound identification was achieved by comparing retention times, mass spectra, and transition patterns between the compounds detected in the samples and those of the reference standards. Both identification and quantification relied on the primary transition observed in the mass spectra of each metabolite. Calibration curves, with correlation coefficients (R^2^) ranging from 0.9964 to 0.9999, were constructed for each metabolite and reference compound to enable quantification. Method validation was performed by evaluating linearity, precision, and accuracy in accordance with International Conference on Harmonization (ICH) guidelines. Limits of detection (LOD) and quantification (LOQ) were established by analyzing a series of different concentrations for each compound. Method accuracy was assessed in duplicate through recovery experiments, and all samples were analyzed in triplicate [46,47,48].

### 4.8. Cytotoxicity Assays

The cytotoxicity of *Betula* spp. extracts was evaluated using HepG2 cells, a human hepatocellular carcinoma cell line, and LX-2 cells, an immortalized human hepatic stellate cell line. Cells were maintained under standard culture conditions, and cell viability was assessed using the Cell Counting Kit-8 (CCK-8) assay. To prepare cell suspensions, cells were detached using 0.25% trypsin–EDTA and centrifuged at 1500 rpm for 5 min. Subsequently, 1 × 10^5^ cells/well were seeded into 96-well plates in 200 μL of complete culture medium and incubated for 24 h to allow cell attachment. Following incubation, *B. pendula* and *B. pubescens* leaf extracts were added at five different concentrations. For *B. pendula*, the tested concentrations were C1 = 188.1 µg/mL, C2 = 359.1 µg/mL, C3 = 515.2 µg/mL, C4 = 658.3 µg/mL, and C5 = 790.0 µg/mL. For *B. pubescens*, the corresponding concentrations were 148.1, 282.7, 405.7, 518.3, and 622.0 µg GAE/mL. The concentrations were selected based on total phenolic content (TPC), expressed as µg GAE/mL. Consequently, the absolute concentrations differed between the two extracts, and the C1–C5 designations represent corresponding concentration levels within each extract rather than identical nominal concentrations between *B. pendula* and *B. pubescens*. Untreated cells served as the negative control, while cells treated with 70% *v*/*v* ethanol in water were used as the positive/internal control. Each experimental condition was performed in triplicate. After 24 h of treatment, 20 μL/well of CCK-8 solution (Sigma-Aldrich, St. Louis, MO, USA) was added and incubated for 3 h at 37 °C. Absorbance was measured at 450 nm using a BioTek Synergy 2 microplate reader (Agilent, CA, USA). Cell viability was expressed as a percentage relative to untreated control cells, calculated as the ratio of absorbance between treated and control cells × 100. Dose–response curves were generated using nonlinear regression analysis [45,49]. IC_50_ values were considered experimentally determinable only when the tested concentration range encompassed a 50% reduction in cell viability. When cell viability remained above 50% across the entire tested concentration range, the IC_50_ was considered to be above the highest concentration tested and was not extrapolated beyond the experimental concentration range [45,49].

### 4.9. Hepatoprotective Activity Assays

#### 4.9.1. Animal Studies

Healthy female Swiss mice (6 months old, mean body weight 32 ± 2.63 g) were obtained from the animal facility of the University of Agricultural Sciences and Veterinary Medicine, Cluj-Napoca. The animals were housed in polypropylene cages (2–5 animals/cage) under controlled environmental conditions (25 ± 1 °C, 55 ± 5% relative humidity, 12 h light/dark cycle), with free access to standard diet and water. Animals benefitted from environmental enrichment, including polycarbonate nests and cardboard tubes. They were clinically evaluated and acclimatized for 7 days prior to the start of the experiment. All experimental procedures were performed in accordance with the European Directive 2010/63/EU and national legislation (Law no. 43/2014) on the protection of animals used for scientific purposes. The experimental protocol was approved by the Institutional Bioethics Committee (approval no. 68/30.05.2017) and authorized by the Veterinary Sanitary and Food Safety Authority (authorization no. 73/14.06.2017). The primary outcome measure for this study was ALT activity, which was used to determine the sample size. We determined the minimum sample size using G*Power 3.1 software. Based on Cohen’s effect size derived from reference data, a minimum of 4 animals per group was required to achieve a statistical power of 0.80 and a significance level of 0.05. The experimental unit was considered the animal; the distribution of animals into experimental groups was balanced based on initial body weight, ensuring that the average weight did not differ significantly between groups. To minimize potential confounding variables, cage locations on the holding racks were systematically rotated weekly to eliminate microenvironmental bias. Furthermore, the order of treatments and subsequent measurements was fully randomized each day. All laboratory evaluation, histopathological assessment and statistical analyses were performed by investigators blinded to the group assignments. Acute oral toxicity was assessed first according to OECD Guideline 423 (Acute Toxic Class Method) [50]. The tested extracts were administered orally at a limit dose of 2000 mg/kg body weight, divided into three administrations given at 4 h intervals in order to not exceed 1% body weight/administration. Acute oral toxicity was assessed in a separate cohort of 10 Swiss mice (5 males and 5 females), distinct from the 35 female mice subsequently included in the 6-week hepatoprotective study. Animals were monitored continuously for the first 4 h post-administration and twice daily thereafter for 14 days. Several human endpoints were considered, including clinical signs of severe or irreversible toxicity, a body weight loss of ≥20%, persistent lateral recumbency, total unresponsiveness to external stimuli, severe respiratory distress, or a body condition score (BCS) ≤ 2. However, no mortality or treatment-related clinical signs of toxicity were observed throughout the study period. Body weight evolution and general behavior remained within normal limits for all animals. At the end of the study, animals were anesthetized with isoflurane, blood samples were collected for biochemical evaluation, and further, euthanasia was performed by cervical dislocation under deep narcosis. Necropsy was performed; gross evaluation revealed no pathological changes. For the hepatoprotective study, a total of 35 mice were divided into seven groups (n = 5/group): reference value (vehicle only), toxic control (CCl_4_ 1.0 mL/kg in vegetable oil), CCl_4_ + *B. pendula* 50 mg/kg, CCl_4_ + *B. pendula* 200 mg/kg, CCl_4_ + *B. pubescens* 50 mg/kg, CCl_4_ + *B. pubescens* 200 mg/kg, and CCl_4_ + chlorogenic acid 50 mg/kg. Hepatotoxicity was induced by oral administration of carbon tetrachloride (CCl_4_, 1.0 mL/kg, diluted in vegetable oil), three times per week (days 1, 3, and 5), for 6 weeks. The normal control group received the same vegetable-oil vehicle used for CCl_4_ administration, administered by oral gavage on the same schedule as the experimental groups. The tested metabolites were administered orally on the same days, 4 h after CCl_4_ administration. Oral administration of all substances was performed using a flexible polypropylene oral dosing tube (Instech FTP-20-38, 20 G; Instech, Plymouth Meeting, PA, USA). The body weight evolution was monitored on a weekly basis. Animals were monitored daily for clinical signs of systemic toxicity and liver failure. The human end-point protocol stated that mice should be humanely euthanized prior to the scheduled end of the study if they met any of the following criteria: a body weight loss of ≥20%, a body condition score (BCS) ≤ 2, persistent hunched posture indicating abdominal pain, visible jaundice (icterus) of the skin or mucous membranes, severe abdominal distension (ascites), or total unresponsiveness to external stimuli. At the end of the experimental period of 6 weeks, animals were anesthetized with isoflurane, blood samples were collected from the retro-orbital sinus, blood samples were collected into 0.5 mL Lithium Heparin microtainers with green caps (Prima; Vetro Design, Bucharest, Romania). The tubes were filled precisely to the volume mark to ensure the correct anticoagulant-to-blood ratio and inverted gently 8–10 times to prevent microclot formation. Euthanasia was performed by cervical dislocation under deep narcosis immediately after blood collection. Liver tissues were collected for further analysis [43,51].

#### 4.9.2. Biochemical and Oxidative Stress Analyses

Blood samples were centrifuged at 1500× *g* for 15 min, and the obtained plasma was stored at −20 °C until analysis. Plasma samples were analyzed using the Element RC Liver Rotor on an Element RC chemistry analyzer (scil animal care company GmbH, Viernheim, Germany). The rotor quantitatively measured a comprehensive liver panel consisting of albumin (ALB), alkaline phosphatase (ALP), alanine aminotransferase (ALT), aspartate aminotransferase (AST), cholesterol (CHOL), gamma-glutamyl transferase (GGT), bile acids (BA), total bilirubin (TBIL), and total protein (TP). A 100 μL volume of lithium heparin plasma was loaded into the rotor, and the automated panel was executed via absorption spectroscopy at controlled parameters over a 12 min cycle. Oxidative stress parameters were evaluated in liver tissue homogenates. The activity of superoxide dismutase (SOD), catalase (CAT), and glutathione peroxidase (GPx) was determined using commercial assay kits (BioVision, Milpitas, CA, USA), following the manufacturer’s protocols. Lipid peroxidation was assessed by measuring malondialdehyde (MDA) levels using the thiobarbituric acid reactive substances (TBARS) assay [43,51].

#### 4.9.3. Histopathological Evaluation

Liver samples were fixed in 10% neutral-buffered formalin, processed by routine histological techniques, and embedded in paraffin. Sections of 5 μm thickness were obtained and stained with hematoxylin and eosin. Histological examination was performed using an Olympus BX 41 microscope (Olympus Corp., Tokyo, Japan). Image acquisition was performed using an Olympus UC30 digital camera (Olympus Corp., Tokyo, Japan) and OLYMPUS Stream Basic software 2.4 (Olympus Corp., Tokyo, Japan). The evaluation focused on hepatocellular degeneration, necrosis, inflammatory infiltrate, and structural alterations of the hepatic parenchyma [43,51].

### 4.10. Statistical Analysis

Data are expressed as mean ± standard deviation (SD). The normality of distribution was assessed using the Shapiro–Wilk test. Differences between groups were analyzed using one-way analysis of variance (ANOVA), followed by Tukey’s post hoc test for biochemical and oxidative stress parameters and two-way ANOVA followed by Bonferroni post-test for body weight variation. Statistical analysis was performed using GraphPad Prism 5 (GraphPad Software, San Diego, CA, USA). The value of *p* < 0.05 was considered statistically significant.

## 5. Conclusions

The findings of this study demonstrate that the leaves of *B. pendula* and *B. pubescens* are valuable sources of polyphenolic compounds with significant antioxidant and hepatoprotective properties. The *B. pubescens* extract showed higher TFC, quercitrin, hyperoside, naringenin, and apigenin content and a stronger antiproliferative effect on HepG2 cells, while *B. pendula* showed higher TPC, stronger DPPH/FRAP antioxidant activity, and better in vivo hepatoprotection/histopathology. Both extracts showed a favorable safety profile toward normal hepatic cells while exerting inhibitory effects on hepatocellular carcinoma cells, suggesting selective biological activity. The hepatoprotective effects observed in vivo further support the potential of *Betula* spp. leaf extracts as natural sources of bioactive compounds for the prevention of oxidative-stress-related liver disorders. Overall, these findings provide a scientific basis for the further investigation of *Betula* species as promising candidates for the development of antioxidant and hepatoprotective phytopharmaceuticals. Further studies are warranted to elucidate the molecular mechanisms underlying these biological effects, identify the key active constituents responsible for hepatoprotection, and evaluate the efficacy and safety of standardized *Betula* leaf extracts in preclinical and clinical settings.

## Figures and Tables

**Table 1:** Evaluation of phenolic content and antioxidant activity of *B. pendula* and *B. pubescens* leaf extracts.

Samples	TPC (mg GAE/g dw)	TFC (mg RE/g dw)	CADC (mg CAE/g dw)	DPPH (IC_50_ mg/mL)	FRAP (mM TE/g dw)
*B. pendula*	39.5 ± 0.07 ^b^	10.6 ± 0.044 ^b^	10.8 ± 0.08 ^a^	0.23 ± 0.06 ^c^	1.784 ± 0.073 ^d^
*B. pubescens*	31.1 ± 0.19 ^a^	29.7 ± 0.02 ^a^	11.1 ± 0.06 ^a^	0.69 ± 0.51 ^c^	1.078 ± 0.08
Trolox	-	-	-	0.012 ± 0.20	-

*Notes*: Values are expressed as mean ± SD (n = 3). GAE = gallic acid equivalents; RE = rutin equivalents; TE = Trolox equivalents; TPC = total polyphenolic content; TFC = flavonoid content; lowercase letters in the same column indicate significant differences (*p* < 0.05); for TPC and TFC, *B. pendula* and *B. pubescens* are significantly different (different letters: ^b^ vs. ^a^) and for CADC, there are no significant differences (same letter “^a^”). Both samples exhibited significantly lower antioxidant activity than Trolox (^c^ *p* < 0.001—Trolox vs. *B. pendula* and *B. pubescens*). ^d^ *p* < 0.001 (*B. pendula* vs. *B. pubescens*).

**Table 2:** Phenolic compounds identified and quantified (µg/mL) in *Betula* spp. extracts by LC/MS/ESI.

Metabolites	Retention Time (Min)	*m*/*z* and Main Transitions	MRM	*B. pendula* Leaves	*B. pubescens* Leaves
*Trans-p* coumaric acid	17.5	163.0 > 119.0	Negative	4.15 ± 0.06	<LQ
Salicylic acid	23.5	137.0 > 93.0	Negative	2.80 ± 0.17	1.53 ± 0.06
Chlorogenic acid	11.9	353.0 > 191.0	Negative	1.42 ± 0.63	0.28 ± 0.02
Neochlorogenic acid				0.52 ± 0.01	0.15 ± 0.13
Cryptochlorogenic acid				8.69 ± 0.31	9.91 ± 0.81
Caffeic acid	13.8	179.0 > 135.0	Negative	0.60 ± 0.01	0.45 ± 0.51
Ferulic acid	18.4	193.0 > 134.0	Negative	1.81 ± 0.02	0.81 ± 0.05
Gallic acid	7.0	168.9 > 125.0	Negative	6.73 ± 2.27	7.91 ± 0.96
Tilianin	20.2	447.1 > 285.0	Negative	2.55 ± 0.09	0.4 ± 0.01
Isoquercitrin	17.6	353.1 > 173.2	Negative	28.68 ± 3.82	24.84 ± 0.72
Kaempferol	27.9	285.0 > 187.0	Negative	1.01 ± 0.01	1.38 ± 0.02
Naringenin	26.2	271.0 > 119.0	Negative	0.39 ± 0.03	1.58 ± 0.01
Quercitrin	22.1	447.0 > 229.9	Negative	0.93 ± 0.41	29.83 ± 0.84
Luteolin—7-*O*-glucoside	19.9	447.0 > 284.9	Negative	0.03 ± 0.01	0.15 ± 0.01
Apigenin	28.1	269.0 > 117.0	Negative	0.21 ± 0.01	2.96 ± 0.08
Quercetol	25.4	300.9 > 151.0	Negative	0.98 ± 0.07	0.17 ± 0.37
Hyperoside	20.3	463.1 > 300.0	Negative	10.36 ± 0.20	15.07 ± 0.06

*Note*: <LQ—below quantification limit.

**Table 3:** Cell viability and estimated IC_50_ ranges of HepG2 and LX-2 cells treated with *Betula pendula* and *Betula pubescens* leaf extracts.

Cell Line	Extract	Concentration Range (µg/mL)	Viability Range (%)	IC_50_
HepG2	*B. pubescens*	148.1–622.0	71.0–80.6	>622.0 µg/mL *
HepG2	*B. pendula*	188.1–790.0	84.9–100.2	>790.0 µg/mL *
LX-2	*B. pubescens*	148.1–622.0	93.1–104.4	>622.0 µg/mL *
LX-2	*B. pendula*	188.1–790.0	95.9–109.5	>790.0 µg/mL *

* IC_50_ was not reached within the tested concentration range; therefore, the reported value represents the lower boundary based on the highest concentration tested and should not be interpreted as an experimentally determined IC_50_.

**Table 4:** Concentration gradient of the mobile phase used in the LC/MS/ESI method.

Time (Min)	Methanol (A)	0.1% Formic Acid in Water (C)
0.00	5	95
3.00	25	75
6.00	25	75
9.00	37	63
13.00	37	63
18.00	54	46
22.00	54	46
26.00	95	5
29.00	95	5
30.00	5	95
36.00	5	95

## Data Availability

The original contributions presented in the study are included in the article, further inquiries can be directed to the corresponding author.
